# Building Capacity for Behavioral Health Services and Clinical Research in a Rural Primary Care Clinic: A Case Study

**DOI:** 10.3934/publichealth.2014.2.60

**Published:** 2014-04-11

**Authors:** Laura M. Daniels, Kim E. Dixon, Lisa C. Campbell

**Affiliations:** 1 Department of Psychology, East Carolina University, Greenville, NC 27858, USA858; 2 Psychology Services, Tuscaloosa VA Medical Center, Tuscaloosa, AL 35404, USA

**Keywords:** depression screening, diabetes self-management, integrated care, behavioral health, rural health, primary care, disparities

## Abstract

Integrating sustainable, evidence-based, and collaborative depression screening and follow-up treatment into primary care clinics is a significant challenge in health care. In this article a case study approach is used to describe the process of building capacity for a depression screening program in a rural federally qualified health center (FQHC). A conceptual framework addressing the clinical, operational, and financial perspectives of a primary care setting is applied restrospectively to identify 1) the barriers and facilitating factors associated with integrating a depression screening program into standard practice and 2) how the program was leveraged to conduct clinical research to improve self-management in patients with diabetes and elevated depressive symptoms.

## 1. Introduction

Assessing for and treating depression in the primary care setting is critical to addressing significant problems associated with comorbidity and premature mortality [1]. According to the U.S. Department of Health and Human Services, healthcare utilization and costs are twice as high in patients with co-occurring depression and diabetes or heart disease [1], [2]. In fact, depression is the most significant contributor to healthcare costs, followed by obesity, arthritis, back/neck pain, and anxiety [3]. Data from a meta analysis of medical cost-offset studies demonstrated a cost-offset when psychological services are offered in outpatient medical clinics and that psychological services are cost-effective as they often pay for themselves with a reduction in medical service utilization among patients with chronic medical conditions and/or psychological distress receiving psychological services [4].

Research has also demonstrated that, in addition to reduction of healthcare costs, treatment of co-occurring depression and chronic conditions has greater effectiveness when care plans are properly designed and implemented using integrated care (IC) models [5]. IC refers to the range of healthcare models that use a systematic approach to care where behavioral health providers (BHP) and primary care providers (PCP) collaboratively create treatment plans, provide clinical services, and coordinate care to meet the medical and behavioral needs of patients [6]. IC clinically and significantly improves health behaviors and biopsychosocial outcomes in patients with co-occurring chronic conditions such as, depression, heart disease, diabetes, and chronic pain [7]–[9].

The primary care clinic is the most accessible and cost-effective setting in which to address depression and its link to physical health concerns using an IC model. About half of patients with mental health concerns visit their PCP before they seek mental health treatment elsewhere in the community [10]. Yet, despite substantial evidence, clinical researchers and healthcare organizations struggle with translating IC research into practice [9]. This problem has been termed the *translation gap,* which refers to the failure in translating efficacious interventions into effective clinical or community services [11].

Rural Federally Qualified Health Centers (FQHCs) are at particular risk of falling into the translation gap. FQHCs are non-profit entities that were established to provide accessible, affordable health care to medically underserved individuals. Notably, FQHCs have recently increased their efforts to provide patient-centered IC [12]. One of the recent goals of the Bureau of Primary Health Care and its partners include reducing health disparities in FQHC populations by improving behavioral health (BH) services, such as mental health screening. However, in low resource settings like FQHCs, BH services are often initially supported by federal, state, and foundation grants, but are not sustainable when the grant funding ends. The problem of low sustainability of programs designed to enhance IC in primary care settings has gained increased attention, but much work remains to be done. To better understand implementation practices and identify critical components of IC, the Agency for Healthcare Research and Quality called for more research using case studies [13].

The first aim of this paper is to present a case study describing how a depression screening program was implemented and sustained in a rural FQHC despite an evolving organizational environment and economic constraints. This case study also describes how the screening program provided critical infrastructure to support a pilot intervention study to improve diabetes management in patients with elevated depressive symptoms. The second aim of the paper is to retrospectively evaluate the screening program and pilot study through the lens of a current IC model and highlight the need for more research on translation, dissemination, and implementation of IC and related clinical research in low resource primary care settings.

## 2. Methodology: Case study of a depression screening program at a rural FQHC

### 2.1 Clinical setting and population served

The site of the depression screening program is a primary care FQHC that serves approximately 9000 patients in rural eastern North Carolina. Consistent with the region in which it is situated, the FQHC provides healthcare services to underserved residents, who as a whole report lower educational attainment, are underemployed, and have limited access to adequate mental health and BH services [14]. Specifically, this region consistently reports the lowest income of any region in North Carolina and has a dearth of mental/behavioral health providers at all levels of training [15]. Most clinic patients are African American (48%) or Hispanic/Latino (27%) and are mostly uninsured, with only approximately 27-32% having some type of insurance, primarily Medicaid and Medicare.

### 2.2 Sources of summary data for depression screening

Clinic administrators provided approval for evaluation of the depression screening implementation over a three-year period (2009-2012). The screening program was adopted as a quality improvement (QI) initiative and quantitative data in the form of screening rates were monitored by clinic administrators and aggregated monthly. No demographic information or screening scores of patients who underwent screening during the evaluation period were collected or analyzed individually or in aggregate as part of the evaluation process. Through consultation with the IRB, it was determined that summary data to be provided by administrators did not include collection of personal identifiers and therefore reporting of this data did not require IRB review for expedited or exempt status.

Qualitative data was collected at monthly staff meetings during which clinic administrators, providers, and support staff identified critical events and behaviors that were influential in the implementation of the depression screening program. Individual interviews were not conducted. Similar to many case studies, data collection and analysis occurred in an overlapping fashion as the authors evaluated the data for its accuracy and relevance to the developing narrative [16].

### 2.3 Analysis

The data was analyzed in several steps. First, the qualitative and quantitative data were organized in chronological order. This chronological ordering facilitated our understanding of the narratives and how the process evolved organically [17], [18]. Second, two authors (LD, LC) reviewed the data relevant to the successes and challenges experienced by the clinic staff during the time period when the depression screening program was being implemented and evaluated. Third, the literature on theoretical and evidence-based integrated care frameworks was reviewed in order to identify a model that 1) was generalizable to low-resource settings and 2) had an established lexicon that could be used to evaluate implementation efforts in similar settings. Fourth, the authors evaluated the clinic's integration process by comparing it to the selected model in order to identify the facilitating factors and barriers to integration. Evaluating case study data using an established theoretical framework is a recommended approach for identifying critical and noncritical data [18].

## 3. Results

In the sections below, the development and maintenance of the depression screening program is described over the course of three chronological Phases. In Phase 1, the planning and initial implementation process of the depression screening program is described. Phase 2 discusses how the screening program provided infrastructure for a pilot diabetes self-management program, and how the diabetes program, in turn, served to reinforce the screening program. Phase 3 describes how the screening program was maintained and enhanced despite changes in key personnel and other operational challenges. Finally, these results are evaluated against a model of IC.

### 3.1 Phase 1: Planning and implementation

In response to the need to increase access to BH services in eastern North Carolina, a clinical health psychologist (co-author KD), in collaboration with the medical and clinical administration of the FQHC, sought and received a foundation grant to establish a health psychology training site at the clinic. The training site would allow for doctoral level clinical health psychologists to provide behavioral chronic disease management services while also training doctoral clinical health psychology interns in a rural primary care setting.

Goals for the BH service included providing on-site behavioral chronic disease management that addressed co-occurring chronic diseases and psychological disorders. For patients with severe psychological distress, the BH team facilitated referrals to community mental health providers. The initial clinical task was to develop a formal and systematic process for identifying patients with depressive symptoms that might undermine patients' capacity for chronic disease self-management. Therefore, a proposal to conduct depression screening for every patient presenting for care at the Department of Family Medicine at the FQHC was presented to, and approved by, the clinic's medical staff and CEO.

The approved screening program was informed by guidelines from the North Carolina Center of Excellence for Integrated Care (http://www.ncfahp.org/adult-depression.aspx), which recommends using the Patient Health Questionnaire - 9 (PHQ-9) as the tool for depression screening and monitoring treatment effectiveness [19]. The PHQ-9 is a nine item self-report screening tool assessing diagnostic criteria for depressive disorders, including an item assessing suicide ideation. The total score (out of 27) indicates severity of symptoms (i.e. scores between 5–9 indicate mild severity, 10–14 indicate moderate, 15–19 indicate moderately-severe, and 20–27 indicate severe depression) [20]. The PHQ-9 was selected as the primary screening tool due to its strong psychometric properties, availability in the public domain, and frequent use in primary care clinics. It was also chosen as a screening tool because it is appropriate for low literacy populations and there is a Spanish version which is an important consideration given the substantial number of Hispanic patients seen at the clinic.

The North Carolina Center of Excellence for Integrated Care guidelines also provide a clear protocol for administering the PHQ-9 and explain when to engage in consultation, treatment planning, and follow-up as determined by scores. First, the protocol specifies that Certified Medical Assistants (CMAs) administer the depression screening tool when a patient presents for care and at least annually thereafter. Second, the PCPs carefully review the PHQ-9 score and determine subsequent treatment in the context of the patient's mental health history and current medical illnesses. With scores above the clinical cut-off (10 or greater), an antidepressant should be prescribed and the patient should be seen by the BH team for additional evaluation and treatment. Finally, for treatment monitoring and follow-up, the protocol suggests that the patient returns to the clinic for a minimum of three follow-up visits of which one of these visits is with the PCP within 4 weeks to assess treatment effectiveness and reassess symptoms with the PHQ-9. The long-term goal of screening and treatment is for the patient to achieve remission of depression symptoms as evidenced by a PHQ-9 score less than 5 with no evidence of ongoing functional impairment.

The medical team received a brief orientation to the depression screening program, which included the role of the CMA, scoring guidelines, and conditions for consultation. By specifying the conditions for consultation, the screening program created opportunities for IC that may have not occurred had the program not been in place and had the full support of the clinic administrators. Further, the consultation approach was important for both patients and BHPs. Specifically, consultation addressing depressed mood and its impact on disease management reinforced the idea that the BHP was part of the team. While no-show rates for BH visits remained quite high, providers reported that patients were more likely to return to the clinic for a follow-up BH visit if they met with the BHP during their medical visit.

**Figure 1.**
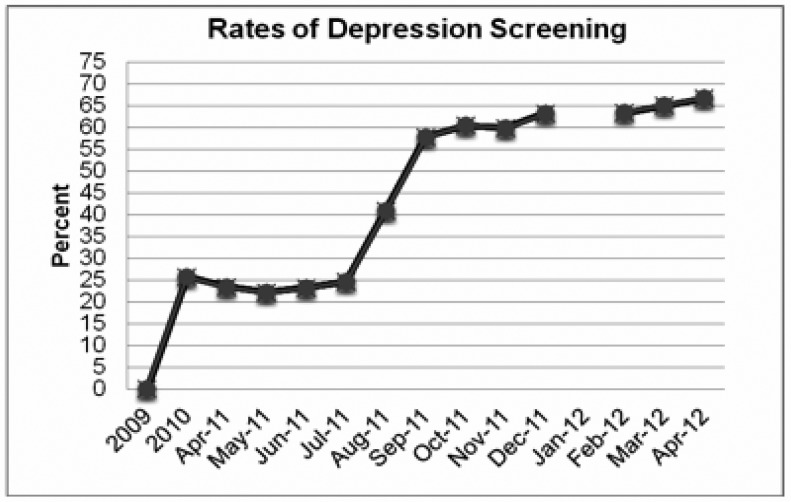
Percentage of patients screened for depression over time and across phases

The electronic medical record (EMR) was an important clinical and operational resource that facilitated implementation of the screening program. To facilitate adherence to the protocol, a reminder flag to screen for depression appeared when the patient's EMR was accessed. The EMR also enabled clinic administrators to monitor the rates of depression screening for inclusion in federally mandated monthly reports. At the conclusion of Phase 1, 25% of the patients seen in the last 12 months had been screened for depression (Figure 1).

Several challenges to implementing the depression screening program should be noted. First, the CMAs encountered challenges in including the administration of the screener into their routine and monitoring patient completion of the screening questionnaire according to the protocol (i.e., monitoring patients during administration to verify literacy and comprehension). The administration of the screener was added to the CMA's already large task list and perhaps it was not a priority to administer the screener and monitor completion of the screener given other tasks competing for their time and attention.

A second challenge related to the PCPs following through on the consultation component of the screening program. After implementation of the screening program, BHPs received screeners with scores above the clinical cut-off, but not until after the patient left the office. Thus, consultation did not always occur during the office visit, as was hoped. Similar to the competing demands experienced by CMAs, the busy schedules and time constraints faced by the PCPs likely resulted in some patients with high depression scores being referred for a later appointment with the BH service, rather than undergoing further evaluation during the clinic visit. Limited availability of the BHPs was also a likely factor in disruptions to the consultation process. The grant that provided support for clinical health psychology internship training only provided for BHP services on site two days per week. This limited availability likely made it difficult for the PCPs to develop a habit of routinely consulting with the BHPs when the screener scores met the clinical cut-off.

When the BHP did not have the opportunity to meet with patients during their medical visits, they typically made follow-up phone calls to check-in and discuss BH services at the FQHC. Anecdotal evidence (co-authors LD, KD, and D. Wallsten, personal communication, n.d) suggested that these phone contacts were moderately effective as many patients agreed to schedule a BH appointment, however, others reported no need for services or that they were “feeling better.” When patients scheduled a BH appointment, no-shows were common which could be due to economic and transportation challenges of having an additional clinic appointment. However, it is also possible that patient uncertainty or stigma related to being seen by a mental health professional was a barrier to keeping the BH appointment. This barrier may have been eliminated if patients had been introduced to the BHP in the course of their medical visit, which could have served to normalize BH care as a routine component of the primary care visit.

### 3.2 Phase 2: Reinforcement of screening program & building capacity for clinical research

The transition into Phase 2 of the depression screening program was marked by two key events: a new goal for screening rates set by the CEO and the receipt of external funding to conduct a pilot clinical research project at the clinic. As noted above, screening rates at the end of Phase 1 were calculated at approximately 25% of patients seen within the past year. In response to this modest rate of screening, the CEO set a new clinic goal to increase screening to at least 50% of patients by the end of the next annual reporting period. To achieve this goal, the clinical team was charged with improving the consistency of screening new patients and annual screening of patients thereafter. Also, the clinic's Quality Improvement Coordinator was assigned the additional task of providing the CMAs a daily list of patients that needed to be screened.

Approximately one year after initial implementation of the depression screening program, one of the co-authors (LC) sought and received funding from the Centers for Disease Control and Prevention to evaluate a pilot program for improving diabetes self-management that would reinforce and expand upon the preexisting depression screening program and clinical health psychology training that had been established at the clinic. The diabetes self-management program was initiated in 2010 following IRB approval.

While the outcomes of that study are beyond the scope of this paper, the synergy that developed between the diabetes self-management program and the depression screening program became an important factor in the success of the screening program. The program consisted of a six-session telephone-based diabetes self-management intervention aimed at providing disease education in conjunction with training in self-management skills and stress and mood management techniques for patients with diabetes who were also experiencing elevated depressive symptoms. The rationale for this program was that patients with diabetes and depressive symptoms evidence poorer glucose control [21]–[22], possibly due in part to the ways in which depressive symptoms can interfere with diabetes self-management (e.g., engaging in consistent self-monitoring, stress management, etc).

At the beginning of the clinic day, the charts of patients being seen for diabetes care were flagged as potential study participants. The note attached to the chart served as reminder that a depression screener should be administered. Patients with diabetes who were eligible for the study had PHQ-9 scores of six or higher, or endorsed the PQH-9 item indicating significantly depressed mood over the past 2 weeks. Ideally, interested patients would meet with BHPs or trainees affiliated with the project, to learn about the project and, if interested, enroll before leaving clinic. Otherwise, interested patients would schedule a return clinic visit to meet with program staff for enrollment and to schedule telephone sessions. The telephone format was chosen for the convenience of the many rural-dwelling patients for whom weekly travel to the clinic for study sessions would be a significant burden. However, patients could opt for face-to-face visits if preferred.

An important feature of the diabetes study that promoted its integration into the clinic was the feedback loop between project interventionists and PCPs. As part of the consenting process, patients gave permission for their interventionist to provide feedback to their PCP or BHP in the event that there was either lack of improvement in or a decline in functioning (e.g., self-management skills remained poor, self-reported blood glucose levels remained high, or patients reported persistent low and/or worsening mood). The providers were very receptive to being part of this feedback loop because it helped them identify patients who were in need of follow-up visits to modify care plans, long before patients were due back for an annual or routine visit.

The study design involved assessing glucose control, disease knowledge, self-efficacy and mood at 3 time points: pre-intervention, immediately post-intervention, and 3 months post-intervention. Study participants received an educational manual, glucometers and a 5-month supply of testing materials free of charge. At the completion of the study, participants also received a 20 dollar gas card. It was recognized that compensation in the form of gas cards would be particularly helpful given that the clinic was situated in a rural region and most patients had limited financial resources and incurred significant transportation costs to access care at the clinic. It was anticipated that there would be patients who declined study participation due to lack of landline or cell phone access or competing life demands. These patients were provided with the educational manual and encouraged to follow up with their PCP.

Importantly, the fact that high numbers of diabetes patients were undergoing screening for depression per the new clinic protocol indicated that the key infrastructure was already in place to identify patients with diabetes who met the criteria for the study. In addition, grant funding for the diabetes self-management program made it possible to leverage the existing BHP presence at the clinic by increasing availability to three days a week. This increased time on-site allowed for supervision of clinical research activities (i.e., seeking referrals for and educating patients about the diabetes self-management program) and expansion of clinical health psychology training activities to include pre-internship doctoral trainees who were supervised in the delivery of the diabetes self-management study.

In Phase 2, the synergy that can exist between clinical and research activities became evident. The existence of a depression screening program that had the full support of clinic administrators and buy-in from clinical staff, enhanced the feasibility of the pilot diabetes self-management study by providing the infrastructure for identification of eligible patients. Once the diabetes self-management study was in place, it reinforced the importance of the depression screening program by demonstrating that, once screened, patients needing additional support for self-management were more readily identified and directed to the resources available through the diabetes self-management study. In contrast, patients who were not screened were less likely to be able to take advantage of the resources available in the self-management study.

### 3.3 Phase 3: Promoting sustainability of depression screening & clinical research feasibility

Phase 3 was a dynamic time at the clinic marked by changes in clinic administrators, turnover in the BHP and their interns, and revision of the depression screening program. Changes in clinic administrators included the loss of the CEO, appointment of an interim CEO, and finally, the hiring of a new permanent CEO. Throughout these changes, the goal to increase depression screening rates remained a priority and significant progress was made. Specifically, CMAs administered the PHQ-9 at higher rates and PCPs made greater use of screening scores by engaging in more in-depth evaluations of depression symptom severity. Subsequently, within a two month period the clinic's screening rate improved to approximately 62%, up from 25% at the end of Phases 1 and 2 (Figure 1).

With regard to BHP turnover, both clinical health psychologists left for other positions and the clinical health psychology internship program closed due to loss of funding during this period (July, 2011). A licensed psychologist (a former trainee of the internship program) was hired as the full-time BHP in October, 2011, to maintain the BH services and oversee the depression screening program. The presence of a full-time BHP promoted more frequent consultation between PCPs and the BHP when warranted by elevated screening scores or other concerns arising during clinic visits. The BHPs increased availability also afforded time for evaluation of any revisions to the screening program.

During this period, the BHP began collaborating with one of the PCPs to revise the screening program based on feedback received during staff meetings. Some of the feedback included the need for clearer screening instructions, problems with administration (i.e. receiving inaccurate screeners; CMAs not verifying literacy), a desire for the PHQ-9 score to be used more consistently as a way to determine when the BHP should be consulted, and a need for a more feasible treatment monitoring plan that could be followed by both the medical team and patients. In response to the feedback, a revised protocol included two key modifications. First, instructions for verifying patient literacy were developed because the FQHC served many patients with limited formal education and literacy. Specifically, the CMAs were to verify literacy by asking patients if they needed assistance in completing the PHQ-9 and, for patients with limited literacy, the PCP verbally administered the screener's first two items (i.e. the PHQ-2) and assessed for suicidality. Second, a more practical timeline was created for follow-up treatment. The earlier version of the protocol called for three follow-up visits with one occurring within 3–4 weeks of the current visit. The revised protocol recommended two follow-up visits, the first to occur within 1-2 weeks to assess side effects, and the second to occur within 6–8 weeks to reassess symptoms with the PHQ-9.

The revised guidelines were distributed and reviewed with the medical team at monthly quality improvement meetings. During the first few months of the new BHP's employment, the rates of screening improved and continued to exceed the clinic goals. At the conclusion of Phase 3, approximately 66% of patients who presented to the clinic had been screened (Figure 1).

While external funding for the internship program and its FQHC-based training site ended, grant funding for the pilot diabetes self-management study continued, reinforcing the importance for depression screening for patients with diabetes and providing PCPs with an important resource for their patients with diabetes who were experiencing depressive symptoms. The pilot study was recently concluded and data analysis and dissemination is under way.

In sum, amidst the changes in the clinic administration, BH service capacity, and revisions to the screening program, the depression screening program was maintained and the screening rates and adherence to the protocol substantially improved. These improvements may be attributed to the fact that screening remained a high priority among clinic administrators, revisions to the screening program addressed the challenges encountered by clinic staff, and the screening program was reinforced by both the presence of a full-time BHP and the highly-visible diabetes self-management pilot study.

### 3.4 Retrospective evaluation of case study through the lens of an IC model

In a review of current IC models, Butler et al. [13] concluded that IC models lead to improved clinical outcomes. There is substantial literature on effective IC models for primary care [6], [23]–[25], methods for integrating BH into primary care [10], and evidenced-based BH assessment measures and interventions for primary care [26]. While current models provide guidelines for what *should* be done when integrating and implementing services, what *actually* occurs in practice can be quite different [27]. This gap between what should occur and what actually does occur is likely greater in low resource primary care environments like FQHCs.

FQHCs are managed within a larger policy and legal environment over which they exert relatively little influence; however, the larger environment shapes much of what occurs in FQHCs. As a result, it must not be assumed that FQHCs are equivalent to other primary care clinics in terms of clinical capacity and operational and financial resources. Few models have been developed and evaluated for their replicability and effectiveness in improving IC in rural FQHCs and other low resource settings. The limited resources and dynamic organizational environment that characterize these settings can create barriers for translating evidence-based IC models into routine practice, thereby increasing the translation gap. Drawing from a review of translational models, the likelihood of improving the quality of care of the healthcare entity increases when: the proposed changes are consistent with evidence-based models, there are resources available to support change, and the environmental influences are identified and managed [28]. In the section below, a model of healthcare quality improvement introduced by Solberg [29] that addresses these elements will be used to highlight the ways in which the depression screening program and pilot diabetes self-management study expanded the FQHC's capacity to implement and sustain improvements in IC.

**Figure 2.**
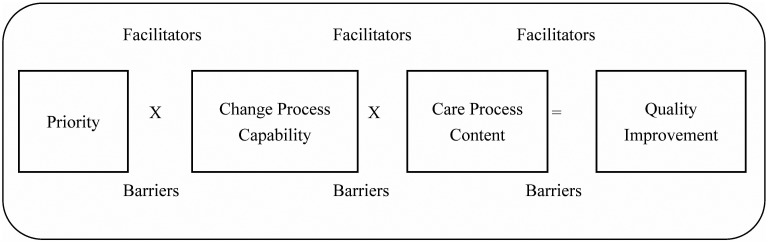
Solberg's conceptual model for practice improvement [29].

Solberg's model for healthcare quality improvement asserts that change is successful when three elements are equally targeted: priority, change process capability, and care process content (Figure 2) [29]. According to this model, change is successful when it is a *shared priority* at all levels of care including the stakeholders, providers, administrative and clinical staff, and patients. The clinic's *change process capability* refers to the preparedness of the clinic and specific strategies and resources that enable and permit change. Finally, quality improvement is successful when the changes to the *content of the* *care process* are evidence-based and standardized. As shown in Figure 2, each of the three elements may be influenced by unique facilitators and barriers; however, all three elements interact with one another to produce or inhibit change. Although Solberg's model has yet to be applied in low resource and rural settings, we chose this model because it makes an important contribution to the BH literature by distinctly clarifying how (i.e. change process capability) the proposed changes are translated into practice. Moreover, it provides a conceptual framework for evaluating buy-in from clinic administrative leadership and investment in change, how valued resources can be reallocated to facilitate change, and how the proposed evidence-based changes may need to be adapted for the local setting.

Implementation and integration of BH services, like mental health screening, is more likely to be successful when there is minimal impact on the clinical, operational, and financial aspects of providing health care [30]. Therefore, in the following retrospective evaluation, we attempt to identify the financial, clinical, and operational facilitating factors and barriers within the three elements of Solberg's model [29].

### 3.5 Priority for change

Priority for change focuses on the importance of getting all levels of care to buy-in to the proposed change, communicating and setting agreed upon goals, and making the changes a high priority on their task list [29]. In this case, an important factor that facilitated routine screening was that depression screening remained a priority and was reinforced, despite changes in clinic administration and BH staff. In Phase 1, administrators made an official announcement of the implementation of the new depression screening program at a staff meeting and the BH team communicated protocol guidelines to the medical team.

In Phase 2, depression screening was reinforced as a high priority by clinic administrators who set a goal to increase screening rates to 50% and enlisted the Quality Improvement Coordinator to facilitate improvements in screening rates. The diabetes self-management pilot study also served to make depression screening a priority in order to ensure that patients with diabetes and elevated PHQ-9 scores that met study criteria would have access to comprehensive diabetes education and glucose testing supplies, should they choose to enroll in the study.

Finally during Phase 3, depression screening clearly became a priority at all levels of care as evidenced by the substantial improvements in screening rates. The CMAs were routinely administering the screener and the PCPs were validating scores and recording them in the patients' EMRs. Further, one of the PCP's collaborated with the new BHP and created a tailored protocol indicating increased commitment and motivation to improve both screening rates and associated treatment procedures.

### 3.6 Change process capability

Change process capability refers to the clinic's state of preparedness for change such as the availability of sufficient resources and strategies for making change happen [29]. Various financial, clinical, and operational resources and effective strategies are needed for improving change process capability [27]. Paired with these resources, strategies that have shown to be successful in creating a system that promotes change include measuring change, providing information and skills training, and creating teams to facilitate change [31].

Throughout each Phase, the allocation of resources and appropriate selection of tools and strategies helped make changes to routine care easier and more feasible. Critical financial resources (i.e. foundation grants) obtained during Phases 1 and 2 provided the impetus to develop a depression screening program and to pilot a diabetes self-management study. In Phase 1, the BHP aptly selected a screening tool, the PHQ-9, that could be quickly completed by patients in a primary care setting and chose an evidence-based protocol that was developed for primary care settings. Notably, depression screening was a shared task such that each member of the clinical team was responsible for a different step in the protocol and not overburdened by the change.

In many ways the EMR was a valuable operational resource used for measuring changes in screening rates. Clinic administrators determined from the EMR's data that additional clinical resources and strategies would be needed to further improve screening rates. In Phases 2 and 3, the EMR was effectively utilized to provide automated reminders to monitor screening rates on a monthly basis. Subsequently, screening goals were set and regular announcements and status updates were made at monthly quality improvement meetings.

Information and skills training were also periodically provided throughout each Phase. During the initiation Phase, the clinical team was trained on how to administer and score the PHQ-9. Also, frequent announcements and reminders of current screening rates, the clinic's goal, and a daily list of patients that needed to be screened were critical information that facilitated the team's engagement in screening. Finally, during Phase 3 the revised protocol was formally reviewed and discussed at the quality improvement meeting which provided initial training to new team members and booster training for others.

Finally, a team-based approach was used to implement the initial screening program, such that staff members at all levels were collectively responsible for carrying out their respective roles in delivering the protocol. This approach created an environment that promoted additional collaborative efforts. For example, the collaboration of the PCP and BHP to create a revised protocol was an example of how teamwork at the provider level was critical for maximizing integration of the screening program into routine care. It is unlikely that screening rates would have increased by 40% during Phases 2 and 3 without strong leadership, flexibility, teamwork, and unique skills that each administrative and clinical staff member contributed during this dynamic time.

The screening program was enhanced by the diabetes self-management study which provided key personnel and patient education resources, as well as strategies for promoting change toward increased integration of behavioral chronic disease management services. As was noted earlier, the pilot study increased the availability of the BHPs by adding an additional day each week during which the BHP and 2 doctoral students engaged in supervised psychological services and diabetes education for self-management. Having these additional personnel on site was important for identifying strategies to promote IC. BHPs monitored the frequency of various staff meetings, the make-up of attendees, and created a schedule for regular updates regarding BH services and the pilot study progress. This served to enhance the visibility of the BH services. Doctoral students assessed aspects of patient flow (e.g., time in waiting room, length of visit) and made recommendations for integrating recruitment and/or diabetes education sessions into the clinic visit with minimal clinic disruption and without burdening patients by lengthening the visit. Strategies for both visibility and integration were well received by clinic staff and administrators.

### 3.7 Care process content

Care process content refers to the evidence-based models and standardized protocols that guide the administrators, clinical team, and staff in making operational and clinical changes [29]. Implementation of the screener during Phase 1 was initiated by the appropriate selection of the evidence-based North Carolina Center of Excellence for Integrated Care depression screening protocol [19]. Similarly, there is substantial literature on the PHQ-9 supporting its use in primary care as a tool for identifying patients with clinically significant depressive symptoms and for monitoring treatment effectiveness.

During Phase 2, the diabetes self-management study was developed according to an empirically supported rationale linking depression management to better chronic disease outcomes. Additionally, the study was conducted according to a protocol that was initially presented to providers at all levels at a monthly staff meeting and reinforced with periodic reviews of procedures and assessment for any changes to clinic patient flow or other operations that would necessitate a change in protocol.

Finally, during Phase 3, adapting the protocol to local clinic operations provided further standardization of each step in the protocol and identified key points of decision making in the screening process for the clinical team. Specifically, the revised protocol included a new standardized process for verifying patient literacy and instructions for administering the screener to patients with very limited literacy levels and/or a language barrier. The treatment monitoring procedure was tailored to improve integration with current clinical operations while still meeting depression treatment guidelines. In sum, the initial implementation and later tailoring of the evidence-based protocol provided a critical framework for developing a standardized patient-centered method for accurately identifying patients with depressive symptoms and referring them for on-site BH services and for possible participation in the pilot clinical research study.

## 4 Implications for the future of IC and clinical research in low resource settings

Through a retrospective case study approach, we examined the process of implementing a depression screening program and leveraging the infrastructure supporting a protocol to conduct a pilot study of a chronic disease self-management intervention. Lessons learned from this examination have important implications for the future of IC and clinical research conducted in low resource settings such as FQHCs.

One implication is that low resource healthcare organizations may be more successful over time by initially focusing efforts and resources on developing the capacity to translate science into practice, rather than waiting for traditional clinical science to reach them. Importantly, the policies that are collectively referred to as “healthcare reform” will increasingly reward healthcare entities that can demonstrate success at delivering integrated medical and BH care as evidenced by lower costs, improved quality, and better health outcomes. Thus, it can be argued that this success will rely, in part, on the organization's capacity to translate promising science into clinical practice that delivers cost offsets, improved quality, and better outcomes. Accordingly, the National Institutes of Health and other major funding agencies increasingly promote translational research that addresses dissemination and implementation science aimed at reducing the 10+ year delay in getting research into practice. Translational research refers to systematic investigations of the process for distributing scientific findings and using effective evidence-based intervention materials with targeted populations [32] and the fidelity of implemented interventions and appropriate adaptations for use in local clinical settings [33]. Those healthcare entities that develop the capacity to partner with researchers and conduct translational research will likely benefit sooner than those that do not.

In the current case study, this capacity grew organically out of an IC setting in which the BHPs also happened to have extensive research training in health psychology. This research training was used to (1) find empirical support for depression as an important factor in chronic disease management that is associated with less favorable outcomes and higher costs; (2) effectively communicate the science to gain buy-in from administrators and clinicians at the local site; and (3) to successfully pursue funding to develop an evidence-based protocol for depression screening that was tailored to the FQHC setting and is now standard practice. In terms of Solberg's model [29], it can be argued that this clinical research skill set was integral for promoting *priority for change*, providing *change process capability* and identifying *care process content.* While much of what occurred in this case was organic, the lessons learned can be intentionally applied in similar FQHC settings by prioritizing research skills in hiring of new PCPs or BHPs, developing these skills in clinicians who are already on staff, or through developing partnerships with clinical researchers in academic settings.

Another important implication is that low resource healthcare organizations may need to develop the capacity to generate original clinical science conducted onsite, such that translation and dissemination occurs simultaneous with efficacy and effectiveness evaluation. Of course, this process is not appropriate for development of new medications or medical devices, but it would be very appropriate for the variety of behavioral medicine and health psychology interventions that are often developed in academic medical center settings and all too often stay there, unless or until they are translated for implementation in lower resource settings. Again, referencing Solberg's model [29], a healthcare organization that has the capacity to conduct original research is essentially creating its own *care process content* which is more readily applied in clinical practice in similar settings as compared to content created in other settings.

More so than the translation and dissemination capacity noted above, the capacity to generate original clinical science would require strong and lasting partnerships between community-based clinics and academic or commercial research entities. These partnerships would afford community clinics the resources to build research infrastructure to attract external funding, particularly funding to develop sustainable programs, rather than programs that require ongoing grant funding.

The diabetes self-management pilot study became feasible due to the successful implementation of the depression screening program and, in turn, served to reinforce the importance of depression screening, demonstrating the potential for synergistic relationships between clinical services and clinical research. The results of the pilot study will be subject not only to academic peer review, but also to administrator and clinician stakeholder review, to identify practices that could have immediate clinical application, or that could be pursued for future clinical application.

A final set of observations relates to the limitations of the approach used in this case study. The quantitative aspect of this study focused only on depression screening rates. Additional quantitative variables could have added additional depth to the narrative. For example, rates of screening broken down by clinical staff or staff categories (e.g., CMAs vs Intern/Trainees vs Physicians) might point to individual or group characteristics that should be reinforced to optimize screening efficiency.

With regard to the qualitative aspects, one could argue for more rigor in the analysis of staff feedback using grounded theory or other iterative approaches to elucidate themes related to adherence or divergence from the screening program. Upon reflection, sacrificing some degree of rigor for feasibility was important to establishing collaborative relationships between clinic staff and administrators and clinical researchers. These relationships were important for developing creative solutions for integrating the more rigorous methods of the diabetes self-management study into the busy clinic environment and can also be leveraged for integration of more rigorous qualitative research in the future.

Finally, while the retrospective application of Solberg's model [29] in the current case study provided a descriptive way to organize a diffuse process, a retrospective approach is limited in its capacity to comprehensively evaluate this model or any others for fit in low resource healthcare environments or to promote model development. Therefore, selection of Solberg's model for descriptive and organizational purposes in this paper should not be construed as an evaluation of this model as being particularly appropriate for the FQHC setting. Ultimately, FQHCs and other community-based clinics that develop the capacity to conduct clinical research will be best positioned to do prospective testing of IC models to evaluate fit and promote future model development.
